# Spatial patterns of mortality in low birth weight infants at term and its determinants in the State of São Paulo, Brazil

**DOI:** 10.1590/1980-549720230034

**Published:** 2023-07-10

**Authors:** Elen Yanina Aguirre Rodríguez, Elias Carlos Aguirre Rodríguez, Fernando Augusto Silva Marins, Aneirson Francisco da Silva, Luiz Fernando Costa Nascimento

**Affiliations:** I Universidade Estadual de São Paulo, Postgraduate Program in Engineering – Guaratinguetá (SP), Brazil.; II Universidade de Taubaté, Postgraduate Program in Environmental Sciences – Taubaté (SP), Brazil.

**Keywords:** Low birth weight, Infant mortality, Spatial analysis, Public health policy, Geographic information systems, Geographic mapping, Baixo peso ao nascer, Mortalidade infantil, Análise espacial, Políticas de Saúde Pública, Sistemas de informação geográfica, Mapeamento geográfico

## Abstract

**Objective:**

Low birth weight (LBW) is a public health problem strongly associated with infant mortality. This study aimed to identify the spatial distribution of infant mortality in newborns with LBW (750–2,500 g) at term (≥37 weeks of gestation), due to their being small for gestational age, analyzing its association with mother-related determinants, as well as to identify priority areas of mortality in the State of São Paulo, 2010–2019.

**Methods:**

Infant mortality rate was analyzed in the division of neonatal mortality and postneonatal mortality of newborns with LBW at term. The empirical Bayesian method smoothed the rates, the univariate Moran index was used to measure the degree of spatial association between the municipalities, and the bivariate Moran index was employed to identify the existence of a spatial association between the rates and the selected determinants. Thematic maps of excess risk and local Moran were prepared to identify spatial clusters, adopting 5% as a significance level.

**Results:**

The excess risk map showed that more than 30% of the municipalities had rates above the state rate. High-risk clusters were identified in the southwest, southeast, and east regions, mainly among more developed municipalities. The determinants of adolescent mothers, mothers over 34 years of age, low education, human development index, social vulnerability index, gross domestic product, physicians, and pediatric beds showed a significant association with the rates evaluated.

**Conclusions:**

Priority areas and significant determinants associated with reduced mortality in newborns with LBW were identified, suggesting the need for intervention measures to achieve the Sustainable Development Goal.

## RESUMO

**Objetivo:**

O baixo peso ao nascer (BPN) é um problema de saúde pública e está fortemente associado à mortalidade infantil. Este estudo teve como objetivo identificar a distribuição espacial da mortalidade infantil em recém-nascidos com BPN (750–2.500 g) a termo (≥37 semanas de gestação), por serem pequenos para a idade gestacional, analisando sua associação com determinantes relacionados à mãe, bem como identificar áreas prioritárias de mortalidade no Estado de São Paulo, 2010–2019.

**Métodos:**

A taxa de mortalidade infantil foi analisada na divisão da mortalidade neonatal e mortalidade pós-neonatal de recém-nascidos com BPN a termo. O método bayesiano empírico alisou as taxas, o índice de Moran univariado foi utilizado para medir o grau de associação espacial entre os municípios e o índice de Moran bivariado foi empregado para identificar a existência de associação espacial entre as taxas e os determinantes selecionados. Mapas temáticos de excesso de risco e Moran local foram elaborados para identificar aglomerados espaciais, adotando-se 5% como nível de significância.

**Resultados:**

O mapa de excesso de risco mostrou que mais de 30% dos municípios apresentaram taxas acima da taxa estadual. Aglomerados de alto risco foram identificados nas regiões sudoeste, sudeste e leste, principalmente entre os municípios mais desenvolvidos. Os determinantes mães adolescentes, mães acima de 34 anos, baixa escolaridade, índice de desenvolvimento humano, índice de vulnerabilidade social, produto interno bruto, médicos e leitos pediátricos apresentaram associação significativa com as taxas avaliadas.

**Conclusões:**

Foram identificadas áreas prioritárias e determinantes significativos associados à redução da mortalidade em recém-nascidos com BPN, sugerindo a necessidade de medidas de intervenção para atingir o Objetivo de Desenvolvimento Sustentável.

## INTRODUCTION

Low birth weight (LBW), defined as less than 2,500 grams, is considered a public health problem, as it is one of the main determinants associated with neonatal, postneonatal, and infant mortality, and the risk of future diseases^1,2^. When LBW is associated with prematurity or fetal growth restriction, the risk of newborn death is higher^3^. Therefore, the lower the birth weight and the lower the gestational age, the higher the risk of death, in difference to LBW newborns with adequate gestational age.

Furthermore, it is estimated that each year more than 20 million newborns worldwide have LBW, which is equivalent to between 15 and 20% of births^4^, and more than 80% of neonatal deaths occur in newborns with LBW, given that they have a higher risk of dying in the first 28 days of life^5^.

In this scenario, the reduction of cases of newborns with LBW has been recognized as a health goal of many countries to reduce infant mortality^6^. In 2012, member states of the World Health Organization (WHO) committed to reducing LBW cases by 30% by 2025^4^, to achieve the Millennium Development Goal of reducing infant mortality by 2015, and the Sustainable Development Goal of reducing neonatal mortality to at least 12 per thousand live births by 2030^7^.

In Brazil, the so-called LBW paradox has been observed, which is characterized by a high prevalence in more developed regions of the country, and lower values in less developed areas. In addition, since 1995 (7.9%) the prevalence trend of LBW has increased^8^. In 2017, the value was 8.5%, and the Brazilian States with a high prevalence of LBW were: the Distrito Federal, Minas Gerais, Rio Grande do Sul, and São Paulo, while the States with the lowest prevalence of LBW were Paraiba, Acre, and Rondonia^9,10^.

Regarding mortality in Brazil, live births with LBW have high rates^9^. Furthermore, low weight is considered a determining factor of infant mortality, especially in the neonatal period, in addition to other determinants related to biological, social, and assistance aspects, such as maternal age and education, among others^11,12^.

Thus, given the efforts of many countries to reduce mortality, in recent years several studies have analyzed cases of LBW and their association with mortality^13–17^. Besides, thanks to geographic information systems (GIS) and spatial distribution studies, it is possible to analyze and apply geographic analytical methods to spatial data for mapping and identifying clusters and risk areas in health problems^18–20^.

Spatial distribution studies carried out with LBW data in different Brazilian States or municipalities showed that risk areas can be identified^14,21,22^, in which a high-priority intervention is important to minimize mortality and LBW in newborns.

In this context, the objective of this study was to analyze the spatial distribution of infant mortality in newborns with LBW at term, and its spatial autocorrelation with maternal-related determinants, as well as to identify risk areas for the reduction of mortality in the municipalities of the State of São Paulo, period 2010–2019.

## METHODS

**Figure 1:**
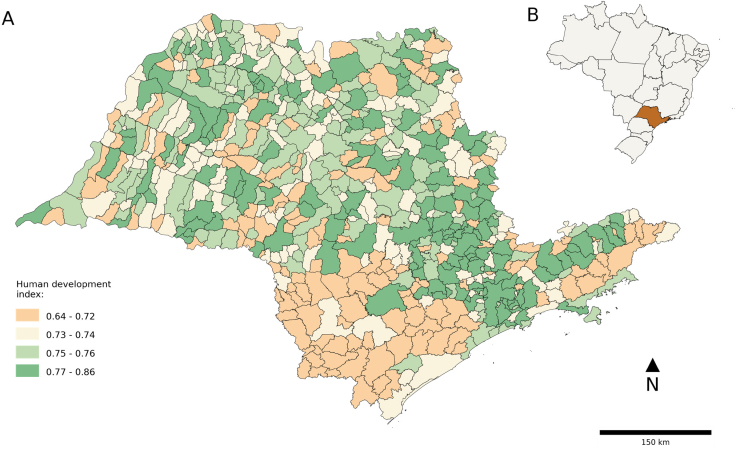
A) Distribution of the Human Development Index. B) State of São Paulo, Brazil. State of São Paulo, 2010.

An exploratory ecological study was carried out based on death data of newborns with LBW at term in the 645 municipalities of the State of São Paulo, between 2010 and 2019, obtained from the Department of Informatics of the Unified Health System (*Departamento de Informática do Sistema Único de Saúde* — DATASUS)^23^. The State of São Paulo has more than 41 million inhabitants registered in the 2010 census, and a human development index (HDI)^24^ equal to 0.78 (high), ranking second in the States (Figure 1), according to the Brazilian Institute of Geography and Statistics (*Instituto Brasileiro de Geografia e Estatística* — IBGE)^25^.

The database for this study included all municipal records of infant deaths with LBW (750–2,500 g) at term (≥ 37 weeks of gestation) from resident mothers, as these babies are considered small for gestational age. In addition, the analysis was carried out in the neonatal (0 to 27 days of life), and postneonatal (28 to 364 days of life) death division, extracted from the Mortality Information System (*Sistema de Informação sobre Mortalidade* — SIM)^23^.

The variables selected for the analysis of spatial autocorrelation related to the mother were: the proportion of mothers under 20 years old, over 34 years old, and low education levels (up to seven years), obtained from the Live Birth Information System (*Sistema de Informação de Nascidos Vivos* — SINASC)^23^. These variables were calculated by the ratio of their number and the total number of live births in each municipality.

The variables related to care were the rate of physicians per thousand inhabitants (considering pediatricians, physicians of the Family Health Strategy, and family and community physicians), and the rate of pediatric beds per thousand live births, collected from the National Registry of Health Establishments (*Cadastro Nacional de Estabelecimentos de Saúde* — CNES)^23^.

The HDI and the social vulnerability index (SVI)^26^ were considered as variables related to the municipality, obtained from the Institute for Applied Economic Research (IPEA)^27^ for the year 2010. The gross domestic product (GDP)^28^ *per capita* per year was obtained from the State Data Analysis System Foundation (*Fundação Sistema Estadual de Análise de Dados* — SEADE)^29^, and the average GDP corresponding to the analyzed period was calculated.

Infant mortality rate (IMR), neonatal mortality rate (NMR), and postneonatal mortality rate (PNMR) per thousand live births with LBW at term were calculated by municipality, using the number of newborns with LBW at term as a denominator.

Crude mortality rates for each municipality were plotted on thematic maps using the quartile category. In addition, a map of excess mortality risk was calculated and generated, which is the relationship between the number of deaths observed in each municipality and the expected number of deaths with LBW in the State^30^.

The empirical Bayesian method was used to calculate the smoothed rates, to stabilize and reduce the high variability observed in the crude rates. The equation for estimating the smoothed rate is given by:


$${\hat{\theta }}_{i}=m_{i}+C_{i}\left(x_{i}-m_{i}\right)$$

Where *m_i_* is the average rate of the analyzed area, *x_i_* the crude rate of municipality *i*, and *C_i_* depends on the population size of the area with values between 0 and 1^31^.

The univariate Moran index (*I_m_*) was used with the smoothed rates to measure the degree of spatial association in the analyzed area, testing the null hypothesis (*H*_0_ = 0) of spatial independence^32^. The *I_m_* is calculated as follows:


$$I_{m}=\frac{n}{\sum_{i}^{n}\sum_{i}^{n}w_{ij}}\times \frac{\sum_{i}^{n}\sum_{i}^{n}w_{ij}\left(x_{i}-\bar{x}\right)\left(x_{j}-\bar{x}\right)}{\sum_{i}^{n}{\left(x_{i}-\bar{x}\right)}^{2}}$$

where *n* is the number of areas, $\bar{X}$ is the overall rate, and *w_ij_* is the spatial weight that reflects the spatial proximity between areas *i* and *j*. The *I_m_* ∈ [–1,1], indicating the existence of an inverse or direct correlation^33^.

The local indicator for spatial autocorrelation (LISA) was used with the univariate local Moran index to identify patterns of spatial association, such as atypical points or clusters^34,35^. The local Moran index can be calculated from the following equation:


$$I_{m}=\frac{\left(x_{i}-\bar{x}\right)\sum_{j}^{n}w_{ij}\left(x_{j}-\bar{x}\right)}{\frac{\sum_{i}^{n}{\left(x_{i}-\bar{x}\right)}^{2}}{n}}$$

Thematic maps were prepared using the Moran map to identify spatial clusters, with the division by categories showing associations according to high-high, low-low, high-low and low-high.

On the other hand, the bivariate Moran index (*I_b_*) was used to identify the existence of spatial autocorrelation between the IMR, NMR, and PNMR with the selected determinants. The bivariate index with a positive value indicates the existence of a positive linear relationship between the values of the two variables, and a negative value indicates a negative linear association. The *I_b_* is calculated as follows:


$$I_{b}=\frac{n}{\sum_{i}^{n}\sum_{j}^{n}w_{ij}}\times \frac{\sum_{i}^{n}\sum_{i}^{n}w_{ij}\left(x_{i}-\bar{x}\right)\left(y_{i}-\bar{y}\right)}{\sqrt{\sum_{i}^{n}{\left(x_{i}-\bar{x}\right)}^{2}}\sqrt{\sum_{i}^{n}{\left(y_{i}-\bar{y}\right)}^{2}}}$$

where *x_i_* and *y_i_* are the two variables analyzed^36^.

The statistical significance level adopted was 5%, and its value was calculated using the Monte Carlo hypothesis test with 999 replications.

Data analysis was performed using the open source programs Python v.3.9.7 and GeoDA v.1.20.0. The study was developed with data made available by the public domains DATASUS, SEADE and IBGE, so it was not necessary to submit the project to the Research Ethics Committee, as established in Resolution n^o^ 510/16 of the National Health Council, of Abril 7^th^, 2016.

## RESULTS

In the State of São Paulo, in the period 2010–2019, 3.5% (212,845) of live births at term had LBW. In addition, there were 68,781 infant deaths in the State, of which 41.0% (28,192) were children with a weight between 750 and 2,500 g, and 11.9% (3,357) were live births with LBW at term. Of these deaths, 1,918 (57.1%) occurred in the neonatal period, and 1,439 (42.9%) in the postneonatal period.

The average IMR in the State of São Paulo in the period 2010–2019 was 15.8/1,000 live births with LBW at term. In the same period, the NMR was 9.0/1,000 live births with LBW, and the PNMR was 6.8/1,000 live births with LBW.

**Figure 2:**
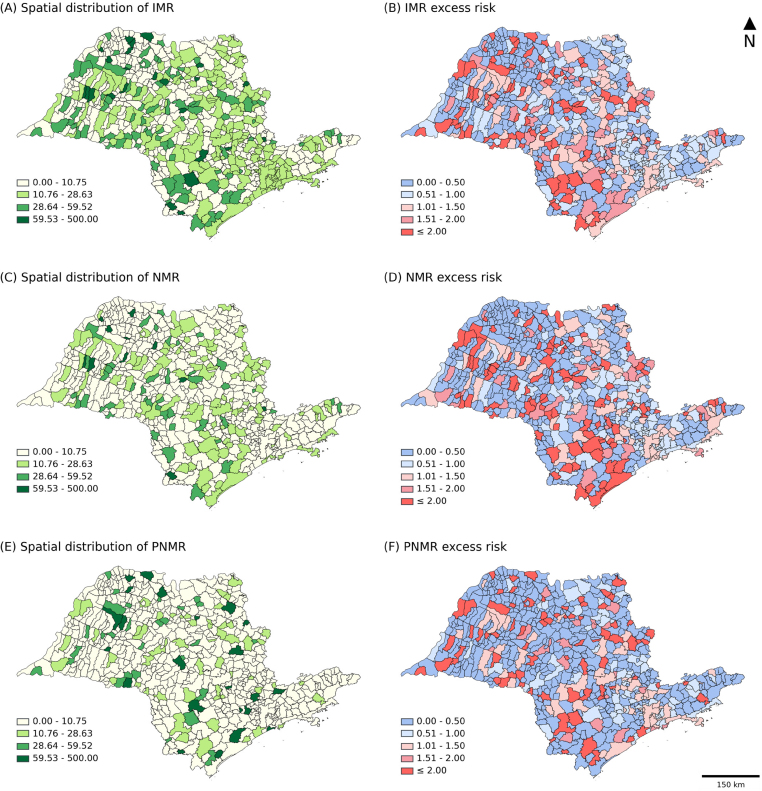
Thematic map of the distribution of the (A) infant mortality rate (IMR), (C) neonatal mortality rate (NMR), (E) post neonatal mortality rate (PNMR), and excess risk of the (B) IMR, (D) NMR and (F) PNMR. State of São Paulo, 2010–2019.

In the analysis of the spatial distribution of the consolidated values for the period 2010–2019, evaluated by the criterion of the quartiles of the IMR (Figure 2A), it was observed that 54.7% (353) of the municipalities had rates below 10.76/1,000 live births, and 28.7% (185) were in the range of 10.76–28.63/1,000 live births. The remaining 16.6% (107) had rates above 28.63/1,000 live births. In the excess risk map (Figure 2B), 33.6% (217) of the municipalities had mortality rates higher than the average mortality rate for the State of São Paulo.

In the NMR (Figure 2C), 74.7% (482) of the municipalities had rates below 10.76/1,000 live births, 17.1% (110) and 6.5% (42) were in the ranges of 10.76–28.63/1,000 live births and 28.64–59.52/1,000 live births respectively, and 1.7% (11) showed values above 59.52/1,000 live births. The distribution of excess risk (Figure 2D) found 198 (30.7%) municipalities with rates above expectations.

The PNMR (Figure 2E) found that more than half (83.9%) of the municipalities (541) had rates below 10.76/1,000 live births, 12.6% (81) had mortality rates in the range of 10.76–59.52/1,000 live births and only 3.6% (23) had values above 59.52/1,000 live births. Furthermore, 149 (23.1%) municipalities had rates higher than those of the State, according to the excess risk map (Figure 2F).

From the spatial autocorrelation analysis, statistically significant spatial clusters (p-value<0.50) were found for the smoothed values of IMR (*I_m_* = 0.09), NMR (*I_m_* = 0.04) and PNMR (*I_m_* = 0.09) in newborns with LBW at term.

**Figure 3:**
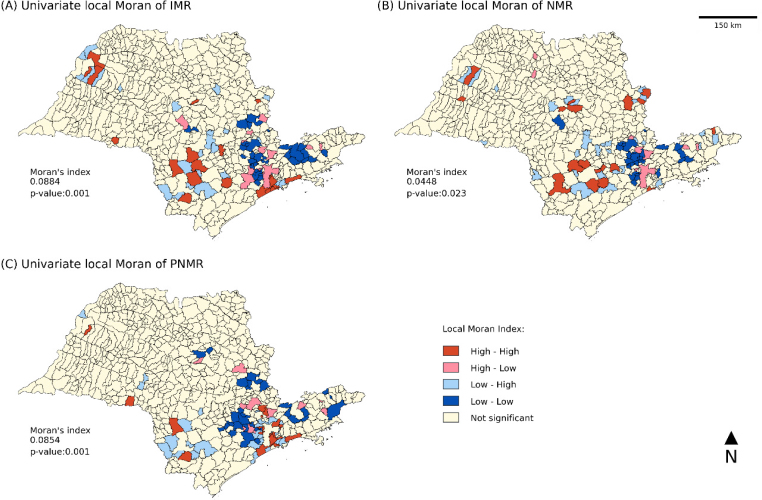
Moran map of (A) infant mortality rate (IMR), (B) neonatal mortality rate (NMR), and (C) postneonatal mortality rate (PNMR) after bayesian smoothing.

Likewise, statistically significant clusters of municipalities were identified, in which 93 municipalities presented significant values in the IMR (Figure 3A), with clusters of high risk rates (high-high) being observed mainly in the northwest, southwest and southeast regions, followed by small clusters of municipalities with high mortality rates surrounded by neighboring municipalities with low values (high-low) in the southwest region. In the central, northwest, southwest and southeast regions, there are small groups of municipalities with lower values surrounded by neighboring municipalities with high values (low-high).

Of the municipalities, 13.5% (87) and 14.0% (90) were significant in the NMR (Figure 3B) and PNMR (Figure 3C), respectively. In the NMR (Figure 3B), small clusters of high-high priority areas were formed in the northwest, southwest, east, and central regions of the state. In addition, close to these clusters there are groups of municipalities with lower values of neonatal mortality (low-high). The southeast region presented clusters of high-low municipalities.

Regarding the PNMR (Figure 3C), high-high and high-low clusters were identified in the southeast and east regions. In addition, groups of low-high municipalities were observed in the southwest and southeast regions. In the three cases analyzed, low-risk clusters were identified in the central, east, and southwest regions.

In the bivariate analysis (Table 1), different degrees of statistically significant association were identified between the analyzed determinants and the smoothed infant, neonatal and postneonatal mortality rates (more details in Supplementary file).

**Table 1:** Bivariate Moran index between infant, neonatal and postneonatal mortality with selected determinants. State of São Paulo, 2010–2019.

Variables	*I*_*m*_ᵃ	Bivariate		
		IMR	NMR	PNMR
Maternal age <20 years (%)	0.34	0.10	0.07	-0.06
Maternal age >34 years (%)	0.30	-0.07	-0.06	0.14
Mothers with low education levels (%)	0.37	0.03	0.04	-0.12
Human development index (HDI)	0.25	-0.06	-0.06	0.11
Social vulnerability index (SVI)	0.33	0.01*	-0.02*	0.13
Gross domestic product (GDP) *per capita*	0.26	-0.06	-0.08	0.11
Physicians per inhabitant	0.12	-0.03*	-0.05	0.21
Pediatric bed rate	0.03	-0.03*	-0.04	0.04

* Not significant (p-value >0.05); (a) *I*_m_: univariate Moran index.

A positive association was identified between the infant and neonatal mortality rates in newborns with LBW at term and mothers under 20 years of age and with low education level. Moreover, a negative association was found with mothers over 34 years old, HDI and GDP. The rate of physicians and pediatric beds showed a statistically significant negative association only with the NMR. The SVI did not show a statistically significant association with infant and neonatal mortality.

The PNMR in newborns with LBW at term showed a different behavior among the associations found. Maternal age below 20 years and low education were negatively associated with postneonatal mortality, while maternal age above 34 years, HDI, SVI, GDP, physicians, and pediatric beds were positively associated.

## DISCUSSION

It should be noted, first of all, that in the literature similar studies of spatial analysis of mortality in newborns with LBW were not found for the 645 municipalities in the State of São Paulo. In this study, through spatial analysis, priority areas of municipalities with high rates of infant, neonatal and postneonatal mortality in term newborns with LBW were indicated, located mainly in the southwest, southeast and east regions, especially risk clusters in more developed cities.

Furthermore, the determinants showed a significant association with IMR, and their results were similar when deaths in the neonatal period were evaluated alone, differently from deaths in the postneonatal period. Thus, the observed risk areas can be included in the design of specific interventions to reduce the mortality and prevalence of LBW.

Infant mortality has decreased worldwide from 1990 to 2015, mainly reflecting the drop in postneonatal mortality, and the number of neonatal deaths has not had the same reduction^4^. These results do not differ much from those observed in some studies in Brazil^37,38^, which show that, between 2011 and 2018, almost three-quarters of neonatal deaths occurred in the first six days of life, and the risk of mortality is even greater for LBW or premature newborns^4^.

In 2017 alone, according to the Ministry of Health report (released in 2019), an IMR equal to 89.0/1,000 live births with LBW was recorded in Brazil, and the State of São Paulo ranked fourth among the states with high LBW prevalence in Brazil^9^. These rates were calculated considering all records of live births and deaths with LBW, including preterm infants.

The findings of this study show that the number of neonatal deaths is greater than the number of postneonatal deaths, and this difference does not change even if only the deaths of newborns with LBW at term are considered. In the State of São Paulo, in the period 2010–2019, about 41% of infant deaths were LBW, more than 11% were newborns with LBW at term, and more than 57% of infant deaths with LBW at term occurred in the neonatal period. Therefore, reducing LBW and identifying its determinants is one of the keys to reducing mortality rates^4,15,16^, as monitoring the municipalities can positively contribute to reducing the number of deaths^4,6,13,38,39^.

In the analyzed period, the State of São Paulo had an IMR equal to 15.8/1,000 live births with LBW at term, in addition to 9.0/1,000 and 6.8/1,000 live births with LBW in the NMR and PNMR, respectively. Apparently, mortality rates are relatively low^14,21^, but there are municipalities with rates above the state rate (above 28.0/1,000). The analysis of excess risk showed that more than 30% of the municipalities had infant and neonatal mortality rates higher than the average mortality rate in the State of São Paulo; in the postneonatal period, the rate was close to 23% of the municipalities. In addition, it was observed that most municipalities with rates above the state average rate are located mainly in the south region, possibly because it is one of the poorest regions of the state^2,29^.

On the other hand, the identification of spatial clusters of the health conditions of live births, as well as the BPN, have been the object of study in recent years. In Brazil, the records of LBW in the 27 federative units were analyzed, identifying high-prevalence risk areas (*I_m_* = 0.27; p-value=0.02) in the south and southeast regions of the Brazilian territory^10^. These regions are the most developed in the country and showed a higher prevalence of LBW, as well as a decreased risk of LBW in adolescent mothers, a higher risk in adult mothers, and a higher risk in mothers with higher education, especially in the north region^35^. In the state of Paraná, no spatial patterns were identified in the prevalence of LBW among its 399 municipalities^40^. In the municipality of Rio de Janeiro, no spatial patterns were identified in LBW (*I_m_* = 0.07; p-value=0.12), showing a random spatial distribution^21^. In the municipality of Salvador, spatial clusters of high NMR (*I_m_* = 0.17; p-value=0.01) were identified in the southwest and central regions, and clusters of LBW prevalence (*I_m_* = 0.19; p-value=0.03) in the north and south regions^14^. Similarly, clusters of high prevalence of LBW were observed in the eastern and western regions of the municipality of Taubaté (*I_m_* = 0.12; p-value<0.01)^22^.

Clusters with high mortality rates are usually associated with several determinants^2,11,12,39,41–43^. Likewise, according to studies, neonatal deaths can be avoided, even if they are newborns with LBW or premature, given a possible association with socioeconomic inequality and the quality of care received by the pregnant woman and the newborn^8,38,41^.

The HDI and GDP showed a negative association with IMR and NMR, indicating that the increase in these determinants in the priority areas would possibly result in a drop in mortality in newborns with LBW^24,26^. The SVI showed no significant association with IMR and NMR. HDI, GDP and SVI were positively associated with PNMR, showing that municipalities with high values of SVI and low values of HDI and GDP will result in lower cases of mortality with LBW in the postneonatal period. This paradox reflects that, even with social inequality, care for newborns in these municipalities is possibly adequate to reduce the risk of death^8,26,28^.

Regarding maternal age, mothers younger than 20 years old or older than 34 years old showed a positive and negative association, respectively, for infant and neonatal mortality. Adolescent mothers and adult mothers are at greater risk of having babies with LBW, and this is related to socioeconomic factors, marital status, occupation, education, etc.^14,39,44^, in addition to emotional and reproductive immaturity in adolescent mothers and aging of the reproductive system in women over 34 years of age.

The mother's schooling can be considered a reflection of the family's socioeconomic level and financial stability, since low values in family income are associated with LBW and the baby's development during pregnancy^24,39,42–45^. Low education level was positively associated with infant and neonatal mortality. The association was negative with postneonatal mortality, possibly due to other factors involved with education not identified in this study.

The rate of physicians and pediatric beds showed a significant association with neonatal and postneonatal mortality, showing the need for improvement in the planning of physical and human resources to enhance newborn care^10,41^. Possibly these results were obtained because many municipalities had low rates of physicians and pediatric beds for the total number of live births.

Finally, the associations identified in the analyzed area make it possible to assess the services and the socioeconomic development of the region^18,40^. The results reinforce what has already been described in other studies^42–44^, suggesting the need for intervention and implementation of health programs and public policies to maintain care for mothers and newborns in municipalities with spatial dependence^46^, with the objective of reversing the negative effects of LBW^16^ and mitigating the risk of infant death.

It should be noted that among the limitations of this work is the use of secondary data made available mainly by DATASUS. Specifically, notifications of infant deaths at term with LBW were obtained from SIM, and the other variables from SINASC, CNES, and SEADE. It was not possible to analyze the dataset in the division of early and late neonatal mortality, as many municipalities were observed with values of zero. Data quality is still affected by incomplete fields^47^; however, the variables used in this study were not affected by this incompleteness. Data collected from the 2010–2019 period were used in a consolidated manner.

In conclusion, priority areas were identified mainly among more developed municipalities located in the southwest, southeast and east regions. Significant associations were found between the determinants analyzed and infant mortality in newborns with LBW at term. In addition, the information produced in this study can contribute to the formulation of intervention strategies for the control and prevention of LBW and mortality in newborns with LBW, among the cities at risk in the State of São Paulo.
